# The Epidemiology of Sleep Quality and Consumption of Stimulant Beverages among Patagonian Chilean College Students

**DOI:** 10.1155/2013/910104

**Published:** 2013-05-16

**Authors:** Juan Carlos Vélez, Aline Souza, Samantha Traslaviña, Clarita Barbosa, Adaeze Wosu, Asterio Andrade, Megan Frye, Annette L. Fitzpatrick, Bizu Gelaye, Michelle A. Williams

**Affiliations:** ^1^Centro de Rehabilitación Club de Leones Cruz del Sur, Punta Arenas, Suiza 01441, Chile; ^2^Department of Epidemiology, Multidisciplinary International Research Training Program, Harvard University School of Public Health, Boston, MA 02131, USA; ^3^Departments of Epidemiology and Global Health, University of Washington, Seattle, WA 98195, USA

## Abstract

*Objectives*. (1) To assess sleep patterns and parameters of sleep quality among Chilean college students and (2) to evaluate the extent to which stimulant beverage use and other lifestyle characteristics are associated with poor sleep quality. *Methods*. A cross-sectional study was conducted among college students in Patagonia, Chile. Students were asked to complete a self-administered questionnaire to provide information about lifestyle and demographic characteristics. The Pittsburgh Sleep Quality Index (PSQI) was used to evaluate sleep quality. In addition, students underwent a physical examination to collect anthropometric measurements. *Results*. More than half of students (51.8%) exhibited poor sleep quality. Approximately 45% of study participants reported sleeping six hours or less per night and 9.8% used medications for sleep. In multivariate analysis, current smokers had significantly greater daytime dysfunction due to sleepiness and were more likely to use sleep medicines. Students who reported consumption of any stimulant beverage were 1.81 times as likely to have poor sleep quality compared with those who did not consume stimulant beverages (OR:1.81, 95% CI:1.21–2.00). *Conclusions. *Poor sleep quality is prevalent among Chilean college students, and stimulant beverage consumption was associated with the increased odds of poor sleep quality in this sample.

## 1. Introduction

Insufficient sleep is a major public health concern and a common medical condition with serious adverse consequences. The recommended durations of sleep are 8.5–9.5 hours for adolescents (10–17 years old) and 7–9 hours for persons ≥18 years of age [1]. Yet many college students do not reach these recommendations and many sleep <6 hours per night. Insufficient sleep has been implicated to affect endocrine, immune, and nervous systems and cardiometabolic risk including obesity, diabetes, impaired glucose tolerance, and hypertension [2]. Additionally, insufficient sleep has been reported as an important factor influencing the regulation of body weight and metabolism [3]. Sufficient sleep enhances memory and has been associated with good academic performance [4, 5]. Sufficient sleep has also been associated with self-rated happiness as was observed in a cross-sectional study of 3,461 Chilean college students [6].

Short sleep duration has been associated with poor academic performance, use of cigarettes, marijuana, and alcohol, mood disorders, physical inactivity, and excessive use of internet [7, 8]. Additionally, investigators have noted that short sleep may contribute to frequent use of medications and alcohol as sleep aids and stimulants use to increase daytime alertness [9]. Consumption of stimulants (e.g., coffee, caffeine shots, and energy drinks) has increased in recent years. Studies have shown that stimulant use among healthy adolescents may be associated with feelings of jitteriness and nervousness, difficulty in sleeping, loss of appetite, and stomach discomfort [10]. Higher doses of energy drink consumption have been implicated in liver damage, chest discomfort, heart rhythm irregularities, increased blood pressure, electrolyte disturbance, kidney failure, heart failure, and death [11, 12]. However, few studies have examined the relationship between energy drink consumption and sleep quality [13, 14].

Since their introduction in 2001, energy drinks have steadily grown in popularity in Chile. Reports indicate that Latin America was the region of the world where energy drinks consumption grew the most, with 31% between 2004 and 2010, while globally the increase was 14.1%. In the same period, energy drinks consumption in Chile increased by 26.7% [15]. These drinks, typically high in caffeine and sugar content, may be particularly appealing to youth and young adults due to marketing efforts. Many energy drink producers advertise their products as performance enhancements for athletics, school, and social situations.

In this cross-sectional study, we assessed sleep patterns and sleep quality among Patagonian, Chilean college students. We also evaluated the extent to which stimulant beverage use and other lifestyle characteristics are associated with poor sleep quality in this population.

## 2. Methods

### 2.1. Study Setting and Sample

This cross-sectional study was conducted at four universities in the Magallanes region of Chile (Patagonia): Universidad de Magallanes, Universidad Tecnologica de Chile (INACAP), Universidad del Mar, and Universidad Santo Tomas between December 2010 and June 2011. Universidad de Magallanes is a research and training university and has more than 160 faculty members and over 4,000 students enrolled in engineering, humanities, social sciences, and health fields. The INACAP is the largest educational community in Chile with 25 campuses from Arica to Punta Arenas, Chile. The Punta Arenas campus has more than 2,000 pre- and postgraduate students. Universidad del Mar is a private institution with about 14,000 students and 15 campuses in 8 regions of Chile. Universidad Santo Tomas is private and has over 18,000 students.

Flyers were posted in each department to invite participants. Students who expressed an interest in participating in the study were invited to meet in a large classroom or an auditorium where they were informed about the purpose of the study and were invited to participate in the survey. Students consenting to participate were asked to complete self-administered individual surveys. There was no time limit for completing the survey. Vision impaired students and those who could not read the consent and questionnaire forms were not eligible to participate. Those enrolled in correspondence, extension, or night school programs were not included as well since their experience might be different from regular time students.

A total of 994 undergraduate students participated in the study. For the present analysis, we excluded subjects over the age of 35 and subjects with incomplete information on sleep quality  (*n* = 162). The final analyzed sample included 832 students (241 males and 590 females). All completed questionnaires were anonymous, and no personal identifiers were collected. Given the minimum risk of the study and use of anonymous questionnaire, waiver of documentation of written consent form was approved by the ethics committees. The procedures used in this study were approved by the institutional review boards of Centro de Rehabilitación Club de Leones Cruz del Sur, Punta Arenas, Chile, and the University of Washington, USA. The Harvard School of Public Health Office of Human Research Administration, USA, granted approval to use the anonymised data set for analysis.

### 2.2. Data Collection and Variable Specification

A self-administered questionnaire was used to collect information for this study. The questionnaire ascertained demographic information including age, sex, and education level. Questions were also included regarding behavioral risk factors such as smoking, energy drinks, caffeinated beverages, and alcohol consumption.

Participants were first asked if they consumed more than one energy drinks or caffeinated beverages per week each month during the current academic semester/quarter. Participants who answered “yes” were then asked to identify the specific type of energy or caffeinated drinks. Energy drinks included international and local brands such as Red Bull, Dark Dog, Burn, Shark, Red Devil, and Battery. Caffeinated beverages included coffee, tea, yerba mate, and cola drinks such as Coca Cola and Pepsi Cola. We summed the number of different stimulant drinks to estimate the variety of different energy drinks or stimulants consumed per week. We use the term stimulant drinks to describe both energy drinks as well as other caffeinated beverages consumed per week.

Sleep quality was evaluated using the Pittsburgh Sleep Quality Index (PSQI) [16]. The PSQI is a 19-item self-reported questionnaire that evaluates sleep quality over the past month. The PSQI yields seven sleep components related to sleep habits including duration of sleep, sleep disturbance, sleep latency, estimates of habitual sleep efficiency, use of sleep medicine, daytime dysfunction due to sleepiness, and overall sleep quality. Each sleep component yielded a score ranging from 0 to 3, with three indicating the greatest dysfunction [16]. Subsequently, the sleep component scores are summed to yield a global sleep quality score (range 0 to 21) with higher scores indicating poor sleep quality during the previous month. Based on the prior literature, participants with a global score of greater than 5 were classified as poor sleepers. Those with a score of 5 or less were classified as good sleepers. This classification is consistent with prior studies of college students [17].

In accordance with PSQI for sleep quality subscales, subjective sleep efficiency, sleep latency, sleep medication use, and daytime dysfunction due to sleepiness, we computed a dichotomous variable of optimal and suboptimal sleep quality. Specific categories were long sleep latency (≥30 versus <30 minutes); estimates of poor sleep efficiency (<85% versus ≥85%); daytime dysfunction due to sleepiness (≥ once per week versus <once a week); and sleep medication use during the past month (≥ once per week versus <once a week). Sleep duration was assessed using the PSQI questionnaire which queried how many hours per night the participants slept during the previous month. Given the lack of prior data on cutoffs for defining “short sleep duration” among college students, we used quartiles. The following quartiles were used to define sleep duration: ≤6.0 hours, 6.1–7.0 hours, ≥7.1–8.0 hours, and >8.0 hours. The group with the lowest quartile of sleep duration (≤6 hours) was defined as having short sleep duration.

We defined alcohol consumption as low (0–4 alcoholic beverages a month), moderate (5–15 alcoholic beverages a month), and high to excessive consumption (≥16 alcoholic beverages a month). Other variables were categorized as follows: age (years), sex, smoking history (never, former, current), and engaging in moderate or vigorous physical activity (no versus yes). Body mass index (BMI) was calculated as weight (kg)/height squared (m^2^). Thresholds of BMI were set according to the World Health Organization (WHO) protocol (underweight: <18.5 kg/m^2^; normal: 18.5–24.9 kg/m^2^; overweight: 25.0–29.9 kg/m^2^; and obese ≥30 kg/m^2^) [18].

### 2.3. Statistical Analysis

We examined frequency distributions of sociodemographic and behavioral characteristics of study participants by quality of sleep. Characteristics were summarized using means (±standard deviation) for continuous variables and counts and percentages for categorical variables. Chi-square test and Student's *t*-test were used to determine bivariate differences for categorical and continuous variables, respectively. The distributions of PSQI scores among male and female students, as well as the sex-specific prevalence of poor sleep quality across age groups, were also estimated. Multivariable logistic regression estimated the odds ratios (OR) and 95% confidence intervals (95% CI) for the associations between poor sleep quality and sociodemographic and behavioral factors in unadjusted and adjusted models. Forward logistic regression modeling procedures combined with the change-in-estimate approach were used to select the final models reported in this research [19]. Prevalence estimates and risk of suboptimal dichotomous sleep quality subscales were also evaluated in relation to stimulant drinks and lifestyle characteristics adjusted for age and gender. All analyses were performed using IBM's SPSS Statistical Software for Windows (IBM SPSS Version 19, Chicago, Illinois, USA). All reported *P* values are two-sided and deemed statistically significant at *α* = 0.05.

## 3. Results

**Figure 1:**
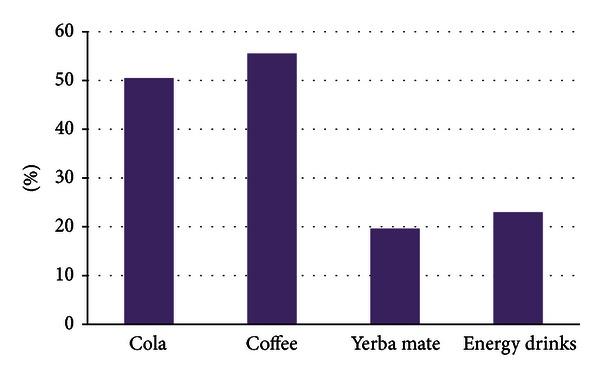
Frequency of stimulant beverages consumed.

Characteristics of the 832 study participants included in the analysis are summarized in Table 1. Approximately 71% of participants were females and the overall mean age was 21.9 ± 3.4 years. Overall, 44.2% of the study population reported being current smokers, 22.5% reported consuming ≥16 alcoholic beverages per month, and 52.0% reported weekly consumption of caffeinated or stimulant beverages. Obesity (14.9%) and physical inactivity (33.5%) were also common in this study population. More than half of the study population (51.8%) was classified as having poor sleep quality. The prevalence of poor sleep quality was higher in females than males for all age groups. Additionally, students with poor sleep quality were more likely to be weekly consumers of stimulant beverages (59.9% versus 49.7%, *P* value = 0.004). The most common types of stimulant beverages consumed were colas and coffee (Figure 1). Approximately 55% of students reported coffee drinking while 50% of them consuming caffeinated cola products (Pepsi/Coke). 

**Figure 2:**
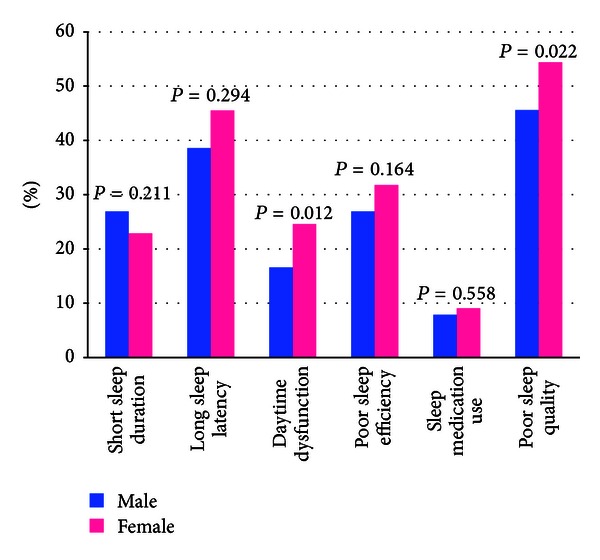
Sleep quality and sleep quality patterns according to gender.

Table 2 summarizes the distribution of PSQI sleep components subscales for the entire study population and for male and female students, respectively. More females (54.4%) than males (45.6%) were found to have poor sleep quality (*P* value = 0.022). Overall, 45.1% of the study cohort reported sleeping ≤6 hours per day. Approximately 41.4% of the cohort reported longer sleep latency (≥31 minutes), and 22.3% reported having daytime dysfunction due to sleep loss at least once per week. A total of 30.4% were classified as having poor sleep efficiency (<85%), and 3.8% reported using sleep medicine at least once per week. Sleep latency was the only sleep quality component that differed by sex, with 81.2% of females versus 71.0% of males (*P* value = 0.007) requiring more than 15 minutes in bed before falling asleep (Figure 2).

We evaluated the odds of poor sleep quality according to participants' demographic and behavioral characteristics. Female students were almost 50% more likely to have poor sleep quality compared with males, after adjusting for all demographic and behavioral covariates listed in Table 3 (OR = 1.48; 95% CI 0.97–2.25), though this association did not reach statistical significance. Adjusting for age and sex, consumers of any stimulant beverages were found to have higher odds of poor sleep quality (OR = 1.52; 95% CI 1.14–2.02) compared with nonconsumers. Further adjustments for cigarette smoking, alcohol consumption, and physical activity strengthened the association. In this larger multivariable model we found that consumers of any stimulant beverage were 80% more likely to have reported poor sleep quality than their counterparts who were nonconsumers (OR = 1.81; 95% CI 1.21–2.73). Physical activity, smoking, and alcohol consumption were not associated with sleep quality in this sample.

**Figure 3:**
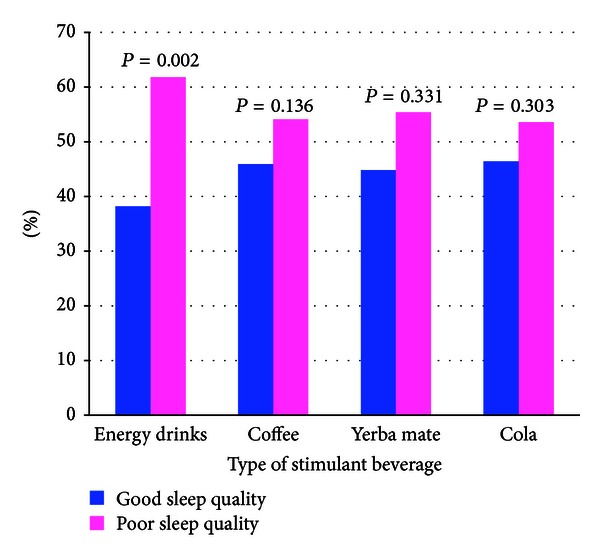
Sleep quality according to types of stimulant beverage consumed.

We next explored the relationships of specific types of stimulant beverages with poor sleep quality. As shown in Figure 3, the prevalence of poor sleep quality was highest among participants who reported consuming energy drinks (including Red Bull, Dark Dog, Battery, Red Devil, Shark, and Turbo Energy) (*P* value = 0.002). Prevalence estimates were lower for participants who consumed coffee, yerba mate, and cola. The prevalence of poor sleep quality was higher among participants who reported consuming three or more stimulant beverages per week (57.9% versus 42.1%, *P* value = 0.073) although this association did not reach statistical significance (data not shown).

**Figure 4:**
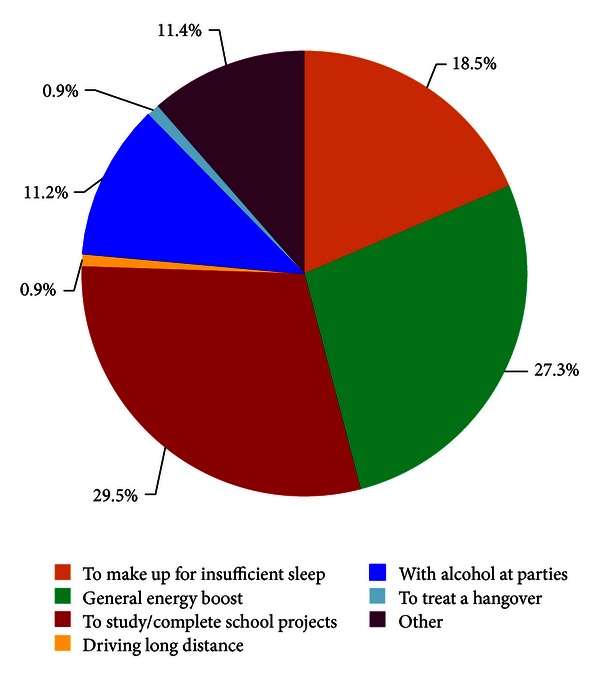
Motivations for consuming energy drinks.

The distribution of stated reasons for consuming energy drinks is summarized in Figure 4. Of the stated reasons for consuming energy drinks, approximately 18.5% of participants reported using energy drinks as a consequence of sleep deprivation, and an additional 27.3% cited energy drink consumption to offset a general need for energy and 29.5% in order to study. Approximately 11.3% of participants reported combining energy drinks with alcohol consumption at parties.

Table 4 summarizes the distribution of stimulant and caffeine use in relation to sleep quality. There was a statistically significant association between poor sleep quality and the consumption of any stimulant beverages (*P*-value < 0.001); those who reported using Red Bull, coffee, and/or tea were more likely to be classified as having poor sleep quality. There was no significant difference in sleep quality between those who consumed coffee/tea with sugar and without sugar.

We next examined the prevalence of specific sleep quality components in relation to demographic and lifestyle characteristics (Table 5). Compared with those who reported never smoking, former and current smokers were more likely to report daytime dysfunction due to sleep loss (OR = 1.72; 95% CI 1.17–2.53) and more likely to use sleep medicines (OR = 2.55; 95% CI 1.40–4.63). Compared with those who consumed <1 alcoholic beverage per month, those who reported consuming ≥16 alcoholic beverages per month had 55% lower odds of poor sleep efficacy (OR = 0.45; 95% CI 0.28–0.72) but increased odds of short sleep duration (OR = 1.42; 95% CI 0.91–2.21), long sleep latency (OR = 1.19; 95% CI 0.77–1.85), daytime dysfunction due to sleep loss (OR = 1.35; 95% CI 0.82–2.23), and increased sleep medication use (OR = 1.19; 95% CI 0.59–2.4)) although statistical significance was not reached. Engaging in any physical activity was associated with reduced odds of short sleep duration (OR = 0.74; 95% CI 0.51–0.96). Any stimulant use was associated with a 50% increased odds of increased daytime dysfunction due to sleep loss (OR = 1.53; 95% CI 1.08–2.18). 

Because energy drinks were more strongly associated with poor sleep quality than other caffeinated beverages, we conducted sensitivity analyses that specifically evaluated odds of subjective sleep efficiency, sleep latency, sleep medication use, and daytime dysfunction due to sleep loss in relation to energy drink consumption. In these analyses we found that students who consumed energy drinks had an 80% increased odds of overall poor sleep quality (OR = 1.83; 95% CI 1.29–2.60) as compared with students who did not use any caffeinated beverages. Students who consumed energy drinks were almost twice as likely to report short sleep duration (OR = 1.93; 95% CI 1.33–2.81) compared with those who did not use any caffeinated beverages (data not shown). The magnitude and direction of associations for other sleep quality parameters were largely similar to those reported in Table 5.

## 4. Discussion

The high prevalence of poor sleep quality among Patagonian Chilean college students is consistent with results from sleep studies among college students in the US and in other countries. We note that consumption of stimulant beverage was associated with increased odds of poor sleep quality and that associations were stronger for those who reported using energy drinks. Although caffeine is the primary ingredient in energy drinks, many include additional ingredients that may have a stimulating effect. The plant Guarana, for example, contains additional caffeine. The amino acid, taurine, a frequent ingredient in energy drinks, is thought to increase the effects of caffeine [20]. Although the absolute amounts of these individual ingredients in energy drinks typically fall below the levels thought to promote adverse effects, little is known about their possible synergistic effects [21].

Available evidence suggests that, when consumed in high amounts or mixed with alcohol, energy drinks may contribute to increased risks of arrhythmia, elevated blood pressures, and psychological symptoms [22]. The stimulatory effect of energy drinks has also been shown to negatively influence sleep quality [23]. Overall, sleep problems are common among college students and are important correlates of significant adverse behavioral and health outcomes including driving while drowsy, reduced cognitive function and productivity, increased interpersonal problems, and lower academic performance [9, 24, 25]. A study conducted in large state university in southeastern United States noted that college students who are at risk for sleep disorders were more likely to have an academic failure (GPA < 2.0) [24]. Another study conducted in the US found that college students with later bed times and wake-up times were more likely to have lower academic performance [26]. The authors estimated that GPAs (provided by the university registrar office) would have been expected to decrease by 0.13 points on a scale of 0–4 for each hour delay in rising time [24, 27]. Possible wellness and health promotion strategies designed to offset these adverse health consequences include circadian rhythm management, sleep hygiene education, and use of sleep stimulants such as white noise [28].

Our results are largely consistent with results from previous studies [29]. For example, in a study of over 40,000 men and women from eight Asian and African countries, investigators reported higher prevalence estimates of sleep problems in female versus male participants. In our study, poor sleep quality was more prevalent among female students. Our findings are also in general agreement with some previous studies that have documented associations of poor sleep quality with increased consumption of caffeinated beverages [30]. For instance, Hindmarch et al. have shown that caffeinated beverages had a dose-dependent negative effect on sleep onset, sleep time, and sleep quality [30]. However, we did not have information concerning frequency and dose of caffeine consumption in the present study to confirm these findings.

Our study has several limitations. First, given the cross-sectional nature of our study, it is difficult to determine whether poor sleep quality is a result of lifestyle factors including stimulant beverage consumption or whether these behaviors resulted as a coping mechanism for the effects of poor sleep. Second, our use of a self-administered survey that relied on subjective measures of sleep quality and other covariates may have introduced some degree of error, and the period of the semester when the survey was administered could have influenced the sleep quality. Third, sleep quality was determined using the PSQI, which relates good sleep quality to global scores of ≤5 and poor sleep quality with scores 6–21. With this broad grouping, there could be substantial heterogeneity among subjects deemed to be poor sleepers, potentially masking important associations. Fourth, we did not have information concerning frequency, timing, and dose of energy drinks consumption in the present study. As a result, it is possible that the binary grouping of energy drinks consumption attenuated the magnitude of association towards null. Fifth, although we adjusted for several potential confounders, we cannot exclude the possibility of residual confounding due to misclassification of adjusted variables or confounding by other unmeasured variables. Finally, our sample was not a random selection which may limit generalizability.

In summary, poor sleep quality is highly prevalent among Patagonian Chilean college students, and consumption of stimulant beverages, particularly energy drinks, is associated with increased odds of poor quality sleep. College students in Chile, and possibly other parts of South America, should be made aware of the impact caffeine beverage consumption has on sleep quality and patterns. Improved sleep quality benefits college students in their daily activities and academic performance and also improves their health status [31, 32]. The college environment and academic demands provide increased exposure to sleep-inhibiting factors, like psychological stress and increased opportunities for social engagements. Avoiding the build-up of a chronic sleep debt during early adulthood through awareness, education, and effective management of sleep disorders may be important in enhancing the academic performance during their college stay and in reducing the development of psychiatric and cardiometabolic disorders later in life.

## Figures and Tables

**Table 1:** Characteristics of the study population.

Characteristics	All	Good sleep quality	Poor sleep quality	
	*N* = 832	*N* = 401	*N* = 431	*P* value
	*n* (%)	*n* (%)	*n* (%)	
Age (mean ± SD, years)	21.9 ± 3.4	21.6 ± 3.2	22.2 ± 3.5	0.231
Age (years)				
18	91 (10.9)	48 (12.0)	43 (10.0)	0.373
19	132 (15.9)	64 (16.0)	68 (15.8)	
20	121 (14.5)	63 (15.8)	58 (13.5)	
21	99 (11.9)	52 (13.0)	47 (10.9)	
≥22	388 (46.6)	173 (43.2)	215 (49.9)	
Sex				
Male	241 (29.0)	131 (32.8)	110 (25.5)	0.022
Female	590 (71.0)	269 (67.2)	321 (74.5)	
Cigarette smoking status				
Never	325 (39.1)	172 (45.6)	153 (38.9)	0.159
Former	77 (9.3)	37 (9.8)	40 (10.2)	
Current	368 (44.2)	168 (44.6)	200 (50.9)	
Alcohol consumption				
Low (0–4 drinks/month)	167 (20.1)	78 (32.5)	89 (32.7)	0.966
Moderate (5–15 drinks/month)	158 (19.0)	73 (30.4)	85 (31.2)	
High (≥16 drinks)	187 (22.5)	89 (37.1)	98 (36.0)	
Weekly consumption of stimulants				
No	356 (42.8)	196 (50.3)	160 (40.1)	
Yes	433 (52.0)	194 (49.7)	239 (59.9)	0.004
Body mass index (kg/m^2^)				
Underweight (<18.5)	10 (1.2)	3 (0.8)	7 (1.7)	
Normal (18.5–24.9)	444 (53.4)	225 (57.8)	219 (52.6)	0.300
Overweight (25.0–29.9)	227 (28.2)	101 (26.0)	126 (30.3)	
Obese (≥30.0)	124 (14.9)	60 (15.4)	64 (15.4)	
Any physical activity				
No	279 (33.5)	140 (38.3)	139 (37.5)	0.826
Yes	458 (55.0)	226 (61.7)	232 (62.5)	

*Due to missing data, percentages may not add up to 100%; physical activity includes moderate and vigorous physical activity.

**Table 2:** PSQI sleep quality patterns by sex.

Characteristics	All	Male	Female	*P* value
	*N* = 832	*N* = 241	*N* = 590	
Sleep duration (hours)				
≤6.0	375 (45.1)	114 (47.3)	261 (44.2)	0.584
6.1–7.0	167 (20.1)	51 (21.2)	116 (19.7)	
7.1–8.0	161 (19.4)	40 (16.6)	121 (20.5)	
≥8.1	129 (15.5)	36 (14.9)	92 (15.6)	
Sleep latency (minutes)				
≤15	181 (21.8)	70 (29.0)	111 (18.8)	0.007
16–30	306 (36.8)	78 (32.4)	228 (38.6)	
31–60	220 (26.4)	64 (26.6)	155 (26.3)	
≥60	125 (15.0)	29 (12.0)	96 (16.3)	
Daytime dysfunction due to sleep loss				
Never	197 (23.7)	66 (27.4)	130 (22.0)	0.066
<once a week	450 (54.1)	135 (56.0)	315 (53.4)	
1-2 times per week	156 (18.8)	34 (14.1)	122 (20.7)	
≥3 times per week	29 (3.5)	6 (2.5)	23 (3.9)	
Sleep efficiency (%)				
≥85	579 (69.6)	176 (73.0)	402 (68.1)	0.431
75–84	127 (15.3)	31 (12.9)	96 (16.3)	
65–74	63 (7.6)	19 (7.9)	44 (7.5)	
<65	63 (7.6)	15 (6.2)	48 (8.1)	
Sleep medicine use				
Not during the past month	758 (91.2)	222 (92.1)	536 (90.8)	0.262
<once a week	41 (4.9)	12 (5.0)	29 (4.9)	
1-2 times per week	17 (2.0)	6 (2.5)	11 (1.9)	
≥3 times per week	15 (1.8)	1 (0.4)	14 (2.4)	
Sleep quality (*a priori* groupings)				
Good	401 (48.2)	131 (54.4)	269 (45.6)	0.022
Poor	431 (51.8)	110 (45.6)	321 (54.4)	

**Table 3:** Odds ratio (OR) and 95% confidence intervals (CI) for poor sleep quality.

Characteristic	Unadjusted OR (95% CI)	Age and sex adjusted OR (95% CI)	Multivariate *adjusted OR (95% CI)
Sex			
Male	1.00 (Reference)	1.00 (Reference)	1.00 (Reference)
Female	1.42 (1.05–1.92)	1.39 (1.03–1.89)	1.48 (0.97–2.25)
Smoking status			
Never	1.00 (Reference)	1.00 (Reference)	1.00 (Reference)
Former	1.22 (0.74–2.0)	1.24 (0.75–2.05)	1.08 (0.55–2.13)
Current Smoker	1.34 (0.99–1.81)	1.26 (0.93–1.71)	0.93 (0.61–1.43)
Alcohol consumption			
Low (0–4 drinks/m)	1.00 (Reference)	1.00 (Reference)	1.00 (Reference)
Moderate (5–15 drinks/m)	1.02 (0.66–1.58)	1.07 (0.68–1.66)	1.15 (0.71–1.87)
High (≥16 drinks/m)	0.97 (0.64–1.47)	1.06 (0.69–1.63)	0.99 (0.61–1.60)
Stimulant beverage consumption			
No	1.00 (Reference)	1.00 (Reference)	1.00 (Reference)
Yes	1.51 (1.14–2.00)	1.52 (1.14–2.02)	1.81 (1.21–2.73)
Physical activity			
No	1.00 (Reference)	1.00 (Reference)	1.00 (Reference)
Yes	1.03 (0.77–1.39)	1.08 (0.80–1.47)	0.96 (0.64–1.43)

*Multivariate includes age and all other covariates listed in the table. For alcohol, the total number of drinks per month was used and the total number of stimulant drinks was also used.

**Table 4:** Consumption of energy drinks, caffeinated beverages and stimulants in relation to sleep quality status.

Exposure	Good sleep quality (*N* = 401)	Poor sleep quality (*N* = 431)	*P* value
	*n* (%)	*n* (%)	
Any stimulant beverages			
No	200 (50.4)	171 (41.2)	<0.01
Yes	197 (49.6)	244 (58.8)	
Type of beverage			
Coke/Pepsi with sugar	178 (44.4)	193 (44.8)	0.910
Coke/Pepsi sugar-free	17 (4.2)	32 (7.4)	0.051
Red Bull	65 (16.2)	108 (25.1)	<0.01
Dark Dog	31 (7.7)	53 (12.3)	0.029
Other energy drinks*	11 (2.7)	22 (5.1)	0.081
Coffee			
No	188 (46.9)	179 (41.5)	0.120
Yes	213 (53.1)	252 (58.5)	
With sugar	140 (34.9)	147 (34.1)	0.807
Sugar-free	73 (18.2)	105 (24.4)	0.031
Tea			
No	328 (81.8)	341 (79.1)	0.331
Yes	73 (18.2)	90 (20.9)	
With sugar	4 (1.0)	5 (0.6)	0.821
Sugar-free	69 (17.2)	85 (19.7)	0.351
Number of stimulant beverages/week			
0	141 (35.2)	121 (28.1)	0.073
1	64 (16.0)	76 (17.6)	
2	106 (26.4)	110 (25.5)	
≥3	90 (22.4)	124 (28.8)	

*Other energy drinks include the following: Burn, Shark, Red Devil, and Battery.

**Table 5:** Prevalence and odds ratios for sleep quality parameters in relation to lifestyle characteristics.

Sleep quality parameters	All (*N* = 832)	Short sleep duration (<6 hours) (*N* = 457)		Long sleep latency (>30 min) (*N* = 345)		Day dysfunction due to sleep loss (*N* = 185)		Poor sleep efficiency (<85%) (*N* = 253)		Sleep medicine use (*N* = 73)	
	*n*	%	OR (95% CI)	%	OR (95% CI)	%	OR (95% CI)	%	OR (95% CI)	%	OR (95% CI)
Smoking status											
Never	325	55.4	1.00 (Reference)	36.6	1.00 (Reference)	16.0	1.00 (Reference)	31.7	1.00 (Reference)	4.9	1.00 (Reference)
Former	77	57.1	1.17 (0.70–1.97)	45.4	1.40 (0.84–2.32)	28.6	2.23 (1.24–3.99)	24.7	0.73 (0.41–1.29)	9.1	1.92 (0.70–4.87)
Current	368	54.6	1.14 (0.83–1.56)	44.0	1.32 (0.97–1.80)	24.7	1.72 (1.17–2.53)	31.2	0.98 (0.71–1.36)	12.2	2.55 (1.40–4.63)
* P* * value for trend *			*0.416 *		*0.100 *		*0.007 *		*0.47 *		*0.003 *
Alcohol consumption											
Low	167	46.7	1.00 (Reference)	38.3	1.00 (Reference)	22.7	1.00 (Reference)	39.5	1.00 (Reference)	10.2	1.00 (Reference)
Moderate	158	55.7	1.50 (0.96–2.37)	40.5	1.11 (0.71–1.74)	23.4	1.09 (0.65–1.84)	28.5	0.61 (0.38–0.97)	8.9	0.90 (0.42–1.91)
High	187	54.0	1.42 (0.91–2.21)	40.6	1.19 (0.77–1.85)	26.2	1.35 (0.82–2.23)	23.0	0.45 (0.28–0.72)	10.7	1.19 (0.59–2.4)
* P* * value for trend *			*0.127 *		*0.89 *		*0.232 *		*0.003 *		*0.85 *
Any stimulant beverages											
No	356	58.4	1.00 (Reference)	37.9	1.00 (Reference)	17.4	1.00 (Reference)	31.2	1.00 (Reference)	8.4	1.00 (Reference)
Yes	433	51.7	0.72 (0.53–0.96)	42.7	1.21 (0.91–1.62)	24.7	1.53 (1.08–2.18)	30.5	0.96 (0.71–1.30)	7.9	0.93 (0.56–1.56)
Physical activity											
No	279	59.1	1.00 (Reference)	39.4	1.00 (Reference)	21.1	1.00 (Reference)	31.2	1.00 (Reference)	8.6	1.00 (Reference)
Yes	458	51.1	0.74 (0.51–0.96)	41.0	1.12 (0.82–1.52)	21.4	1.07 (0.74–1.55)	29.7	0.94 (0.68–1.31)	7.9	0.94 (0.55–1.62)

*Adjusted for age and gender; *all frequencies in the table (except for those of all subjects) indicate percentages within the lifestyle characteristics; ^†^low (0–4 drinks/month); moderate (5–15 drinks/month); high (≥16 drinks/month).
